# Supplementary material for: Surface Microbiome Profiling of Dental Elevators Using Third‐Generation Sequencing: Implications for Infection Control in Dental Practice
Source: Microbiologyopen. 2025 Dec 8;14(6):e70178. doi: 10.1002/mbo3.70178 (PMC12685768; doi:10.1002/mbo3.70178)
Supplement: Supplementary file 1 — customer_backup. [file MBO3-14-e70178-s001.zip › customer_backup/customer_backup/taxa_summary/krona/groups/treat/M17.Krona.html]

Javascript must be enabled to view this page.

members
magnitude
magnitudeUnassigned

M17.krona

55010

54999

81

0

0

0

0

0

0

0

0

0

0

0

0

0

0

0

81

81

81

81

0

27

16

0

0

22

2

0

14

0

0

0

0

0

0

0

0

0

0

0

0

0

0

140

13

0

0

0

0

1

1

0

0

1

0

1

0

0

0

0

0

0

12

0

0

0

0

0

0

0

0

0

0

0

0

0

12

12

12

0

0

0

0

0

0

0

0

0

0

0

0

0

0

0

127

125

0

0

0

125

125

125

2

0

0

0

0

0

0

1

1

1

0

0

0

1

1

1

162

2

2

0

0

0

2

0

0

0

2

1

1

0

0

0

0

43

0

0

0

0

43

0

0

0

0

0

0

0

0

0

0

0

0

0

43

0

0

0

0

0

1

1

0

33

0

8

23

2

5

5

4

4

0

0

0

0

0

0

2

2

2

2

2

0

0

66

66

6

0

0

0

0

0

0

0

0

0

0

0

0

6

6

0

0

0

0

0

0

0

0

0

60

60

4

42

0

14

0

0

20

20

20

0

0

20

20

0

0

0

0

0

0

0

0

0

0

0

0

0

0

0

0

0

0

0

0

0

29

29

29

29

29

0

0

0

0

0

0

10

10

0

0

0

0

6

6

6

6

4

4

4

4

21

21

21

17

17

17

0

0

0

4

4

4

0

0

0

0

0

0

722

254

254

254

17

17

121

121

103

10

93

13

5

8

14

14

14

14

14

454

454

454

100

100

0

341

0

2

190

1

148

13

13

0

0

0

626

173

173

0

0

0

173

173

173

25

25

0

0

0

0

0

0

25

25

25

0

0

0

0

0

428

133

133

1

0

0

1

132

0

48

84

0

0

0

0

0

1

0

0

0

1

1

1

0

0

259

57

6

6

6

6

45

15

30

0

0

0

0

0

0

0

192

7

7

0

185

0

185

0

0

10

10

0

10

35

35

4

4

0

0

0

0

31

0

31

0

0

0

0

0

0

0

29294

20319

0

0

0

0

593

35

35

35

456

6

6

0

45

24

21

30

30

375

1

364

10

102

102

102

173

173

98

5

93

75

75

0

0

21

0

0

0

21

0

0

0

0

0

0

21

21

0

0

0

40

40

29

0

0

29

11

0

0

0

11

18

13

13

13

5

5

5

7565

7565

0

0

253

28

9

82

6

128

0

0

94

94

0

0

197

197

2

2

0

0

0

0

30

0

0

0

10

2

18

0

0

0

1019

0

659

124

236

0

0

0

5779

0

2

0

116

4

0

1

0

2

21

0

21

474

79

21

0

0

1546

1295

15

1851

10

0

20

288

2

5

6

0

0

191

0

7

0

184

16

16

16

16

0

0

0

0

0

3246

222

0

0

4

0

4

11

11

0

0

0

20

19

1

187

54

20

113

27

27

27

323

48

0

48

151

40

20

0

5

0

86

0

0

124

124

0

0

0

0

0

0

0

0

0

7

7

7

0

0

0

991

0

0

9

9

0

0

0

41

41

0

185

185

0

259

259

0

0

0

0

0

14

14

372

17

97

197

2

0

5

54

18

18

90

0

13

0

40

0

0

0

0

0

14

0

23

3

3

103

0

0

8

8

70

70

0

0

25

25

0

0

0

7

7

7

8

8

8

0

0

0

109

5

0

0

5

6

4

2

6

6

92

0

8

46

38

71

52

30

22

19

19

0

277

16

0

16

13

13

33

33

0

0

0

0

0

4

4

40

40

0

0

171

47

124

1

1

0

1

1100

352

352

0

0

0

0

10

9

0

1

4

4

536

3

0

2

3

130

61

106

103

37

4

74

13

5

5

26

26

24

5

19

0

0

0

0

1

1

0

0

142

142

15

15

15

15

0

0

307

0

0

0

0

0

307

161

14

0

92

55

46

4

42

19

0

15

4

0

22

22

59

0

54

5

17

17

17

17

27

27

3

0

0

3

24

0

22

2

0

0

0

0

0

0

0

0

0

0

2

2

2

2

135

135

135

133

2

0

0

0

0

0

0

0

0

0

0

0

1

0

0

0

1

1

1

6498

6498

56

56

41

0

41

6218

6218

22

10

11

0

1

0

0

0

0

0

0

12

5

7

0

0

0

145

94

0

51

4

4

0

0

8

8

8

8

375

40

40

40

0

0

0

7

7

7

4

4

4

241

241

241

0

0

0

0

0

0

0

0

0

83

83

83

1262

1262

0

0

12

12

0

0

0

0

111

111

0

0

0

0

0

0

0

0

0

0

0

0

0

0

0

58

58

0

0

0

5

5

23

0

21

2

0

0

1028

20

463

545

0

0

0

0

25

0

20

5

8975

0

0

0

0

0

8

8

0

0

8

0

8

7

0

0

0

7

7

7

5204

7

1

1

6

6

24

0

0

0

0

24

24

23

9

9

0

0

0

14

14

0

0

0

0

0

0

0

0

0

0

37

37

37

0

0

2702

0

0

0

0

823

823

699

53

1

45

600

1180

0

68

30

9

34

691

54

81

13

25

5

11

45

56

0

58

103

0

0

5

5

59

0

59

0

17

17

0

0

22

0

22

9

9

9

96

0

0

0

0

8

8

70

3

0

67

18

11

7

0

0

0

61

0

0

26

26

35

0

35

0

0

0

0

134

134

134

2006

0

0

6

0

0

6

17

17

30

30

16

16

165

148

0

17

808

111

697

0

0

142

0

142

0

0

102

0

102

209

112

96

1

19

19

109

0

109

383

383

0

0

2

0

0

2

0

2

147

38

29

28

1

9

9

0

0

109

105

23

82

0

0

4

4

1

1

1

1

34

20

20

20

14

14

14

0

0

0

0

0

0

0

0

0

0

0

0

0

0

0

0

0

0

0

0

0

0

0

0

336

1

1

1

19

8

5

3

6

6

0

0

0

0

5

0

0

0

5

0

0

0

0

0

0

0

0

0

0

0

0

0

0

0

0

0

0

0

0

0

298

298

0

0

295

3

13

0

0

0

0

2

2

0

11

7

4

0

0

0

0

0

5

5

5

0

0

0

0

0

0

0

0

0

0

0

0

0

0

0

0

0

0

0

0

0

0

0

8

0

0

0

8

0

0

8

8

0

0

0

0

0

0

0

33

0

0

0

0

0

1

1

1

3

0

0

0

3

3

2

2

2

23

3

3

10

10

10

10

0

0

0

0

0

0

0

0

0

0

0

0

0

0

0

4

2

2

0

0

1

1

0

0

0

0

0

0

0

1

1

0

0

0

0

0

0

0

0

0

0

0

0

16

16

16

0

0

0

0

6

9

0

1

0

0

0

0

0

0

0

0

0

0

0

3000

708

164

164

38

0

38

506

506

0

0

0

0

0

2292

18

18

16

16

0

0

38

38

0

24

0

14

0

10

2196

0

0

620

0

6

0

759

112

43

25

8

37

586

0

0

0

0

0

0

0

0

0

0

0

0

0

0

0

0

0

0

0

0

0

0

68

68

18

12

1

5

0

0

0

0

0

0

9

9

31

4

0

27

10

10

0

0

0

0

0

0

0

0

83

0

0

0

0

0

0

83

83

83

13

13

13

0

13

0

0

17

17

17

17

0

541

399

399

0

0

0

399

26

26

0

0

55

55

69

69

116

18

98

13

13

0

0

120

16

78

26

142

5

0

0

0

5

5

5

20

11

11

11

9

9

9

0

0

0

55

24

0

0

0

8

5

3

0

0

0

16

11

0

5

0

0

0

0

0

0

0

30

4

1

3

26

11

0

15

0

1

1

1

0

0

0

0

0

0

33

33

33

0

0

0

33

0

0

0

0

0

0

0

0

0

0

0

0

0

0

0

29

29

18

18

11

11

84

84

84

84

84

0

32

3

49

10

10

10

10

10

10

2400

0

0

0

0

0

2387

1458

1

1

1

1369

10

10

0

0

0

0

0

29

0

29

0

0

6

0

6

0

0

0

8

8

573

1

12

23

13

23

476

0

0

25

257

0

145

79

33

0

0

0

0

0

34

12

0

22

128

128

0

0

185

68

114

3

139

139

88

88

4

12

72

0

0

0

161

0

0

0

11

0

0

0

0

0

0

0

0

2

2

0

0

0

0

2

2

0

0

0

0

0

0

0

0

0

0

0

0

0

0

0

0

0

7

7

0

0

0

0

0

0

0

0

0

0

0

0

0

0

8

8

8

0

0

142

0

0

0

0

142

142

0

0

279

1

1

1

0

0

12

12

12

0

0

0

146

1

1

6

6

41

41

98

98

13

2

2

0

0

0

0

0

0

0

0

0

0

0

11

0

6

0

0

0

2

0

3

0

0

0

0

0

0

0

0

0

0

0

0

0

0

5

4

4

1

1

0

0

0

46

46

0

6

18

8

2

1

0

0

0

0

4

0

3

0

2

0

2

15

1

1

1

1

0

13

3

1

0

9

0

0

27

0

0

0

17

0

17

3

0

0

3

0

0

3

3

2

2

0

0

1

0

1

0

0

0

0

0

0

0

0

0

0

0

0

0

0

0

1

0

0

0

0

1

0

0

0

0

0

0

0

0

0

0

0

0

0

0

0

0

0

2

2

0

1

0

1

0

0

0

0

0

12

12

12

26

6

6

6

20

6

0

6

0

14

14

0

0

0

0

0

0

0

0

0

0

0

0

0

0

0

463

31

3

3

0

0

6

6

22

5

17

0

10

0

0

0

0

0

0

0

0

10

10

40

0

0

14

14

26

11

15

0

0

0

0

0

0

0

0

62

58

58

0

0

4

4

0

0

320

2

2

153

4

20

0

88

24

17

4

4

0

0

0

3

3

0

158

0

19

11

12

82

12

0

0

7

0

5

0

4

6

0

0

0

0

1

1

1

1

1

0

0

11

11

7

7

7

4

4

4

1

1

0

0

0

0

1

0

0

1

1

0

0

0

0

406

322

5

5

5

5

0

0

0

0

0

0

4

0

0

0

4

0

0

4

4

0

0

0

0

0

0

0

0

313

307

307

307

6

6

6

0

0

0

0

84

84

84

26

26

0

24

24

34

0

34

0

0

0

0

36

36

36

36

36

36

0

0

10880

11

11

1

1

1

8

8

1

0

0

7

0

0

0

0

0

2

2

2

0

0

0

0

0

0

0

0

0

0

0

0

0

0

0

0

0

0

0

0

0

0

0

0

0

0

0

111

111

111

111

111

1972

106

103

0

0

3

3

67

67

1

1

8

8

12

12

12

12

3

3

0

3

215

44

44

44

25

25

25

0

0

0

0

0

0

0

0

0

146

146

146

465

22

22

22

24

24

24

60

60

60

14

14

14

303

303

303

42

42

42

2

2

2

2

1184

119

0

0

0

111

0

111

8

0

4

4

324

324

0

8

316

357

27

0

27

313

0

313

17

17

0

384

0

0

0

0

384

384

2709

1381

12

12

12

652

439

0

101

6

13

137

129

53

0

0

20

20

0

71

0

0

71

92

62

15

15

30

30

717

88

88

5

5

624

624

0

0

0

0

1328

600

600

24

576

0

728

0

0

0

0

728

728

6022

0

0

0

0

6

6

6

6

0

0

0

0

0

0

0

0

1

1

1

1

18

18

2

2

2

2

0

0

0

1

1

0

0

0

0

0

0

0

0

0

1

0

1

0

5

5

7

7

0

0

0

0

11

11

11

5

0

6

0

0

0

0

73

8

1

0

0

0

1

7

7

4

4

0

0

1

3

61

55

2

53

4

4

2

2

0

0

28

28

0

0

0

28

0

17

0

0

0

3

0

8

0

0

277

137

29

29

108

108

0

0

19

2

2

17

17

0

0

0

0

0

0

0

0

0

0

0

121

2

2

25

25

94

94

10

2

0

0

0

2

2

8

5

5

3

0

3

140

140

17

0

0

17

123

93

1

7

0

0

2

6

0

0

2

0

11

1

53

53

53

53

0

0

0

49

49

0

0

49

5

7

11

3

23

0

0

680

168

3

3

2

2

1

1

0

0

0

0

0

0

0

0

0

0

160

0

160

0

2

2

512

29

10

0

19

0

0

445

0

9

10

0

0

2

155

29

10

16

23

25

0

6

83

2

2

8

9

50

0

6

0

5

5

33

2

31

0

0

38

38

2

2

1

1

3

2

1

0

0

0

0

6

0

3

0

0

3

0

19

0

3

1

15

0

0

2

2

5

5

4638

4137

1

1

0

0

491

0

491

0

0

4

4

0

2999

4

1960

46

826

163

0

0

0

0

642

642

14

0

0

4

4

6

6

3

0

3

0

0

0

1

1

0

0

0

0

0

375

346

342

4

0

29

29

84

4

4

0

0

4

4

10

9

1

0

0

0

0

0

0

0

0

0

0

4

4

0

0

8

8

38

0

2

36

16

16

0

0

0

0

0

0

0

0

0

28

27

27

1

1

55

55

55

55

8

0

44

3

4

4

4

4

0

0

2

0

0

0

0

0

0

0

1

0

0

1

0

0

0

0

0

0

0

0

0

1

0

1

1

1

0

0

0

0

0

0

0

0

0

0

0

0

1043

1043

1043

1043

1043

1043

42

42

42

42

2

2

3

3

12

12

25

24

1

0

0

0

0

0

0

0

0

0

0

0

27

27

27

8

8

0

0

8

0

0

3

3

0

3

0

0

0

0

4

4

4

12

12

12

81

4

4

4

4

4

21

21

6

6

6

15

15

15

0

0

56

56

0

0

0

0

0

0

56

56

56

19

19

19

19

19

14

5

0

0

641

206

5

5

5

5

201

80

56

56

0

6

6

0

0

18

18

121

44

44

1

1

0

0

76

76

136

25

25

25

1

20

4

0

3

3

3

3

0

0

0

108

108

0

0

38

0

16

22

25

0

25

27

10

0

17

18

5

0

13

26

0

0

0

0

22

22

22

22

4

4

4

4

80

80

80

4

4

76

0

3

0

0

43

0

0

30

8

8

8

8

8

185

185

17

17

17

167

167

167

1

1

1

0

0

0

0

2216

16

0

0

0

0

3

3

3

3

13

13

13

13

90

10

10

10

10

57

26

26

26

31

31

31

7

7

7

7

7

7

7

7

0

0

0

9

9

9

9

0

0

0

0

17

0

0

0

0

12

12

12

12

5

3

3

3

2

2

2

0

0

0

0

0

0

0

0

2093

2093

1503

1503

1503

29

29

29

0

0

0

45

45

45

0

0

25

25

25

276

0

0

185

0

185

7

7

77

77

7

7

0

0

60

60

60

155

155

155

35

35

35

0

0

0

35

35

35

0

0

0

0

0

0

0

0

0

0

0

0

0

0

0

0

0

0

0

0

183

127

17

17

9

9

8

5

3

5

5

5

5

105

40

40

40

34

34

34

9

9

9

11

11

11

11

11

11

0

0

0

0

0

0

0

0

56

32

32

9

9

23

0

23

24

24

24

4

20

111

111

111

24

24

0

24

0

87

40

7

33

47

15

0

5

5

0

4

18

17

17

17

17

0

0

17

17

4755

1015

0

0

0

0

0

0

0

0

0

0

0

0

0

0

1

1

0

0

1

1

2

0

0

0

0

0

0

2

2

2

0

0

9

9

9

0

9

0

731

731

1

1

0

0

0

0

0

0

0

61

15

46

0

0

0

23

23

0

0

0

0

3

1

2

0

0

0

0

1

1

0

0

0

0

2

0

2

0

13

0

11

2

0

0

7

0

3

0

0

0

0

3

0

0

0

0

0

1

0

0

0

0

0

0

0

0

0

0

0

20

0

12

8

0

14

5

8

1

5

2

0

0

2

0

1

14

0

0

0

0

12

0

0

2

0

0

0

30

0

0

1

29

0

59

59

0

0

0

0

0

0

0

0

0

1

0

1

1

1

2

2

0

0

52

0

17

34

1

0

0

0

0

74

74

1

1

0

0

0

0

0

0

0

0

37

3

34

0

0

0

310

0

182

6

33

0

66

6

3

1

1

0

12

0

0

0

0

0

0

0

0

0

0

72

14

0

0

0

0

0

0

0

0

0

0

0

12

12

0

0

0

2

1

0

0

0

1

0

0

0

0

0

29

3

3

0

0

1

1

0

0

1

1

0

0

24

23

1

29

0

0

0

0

0

0

0

0

0

0

0

29

29

33

33

0

0

6

6

27

26

0

0

0

0

0

1

0

0

12

2

2

2

0

0

0

0

0

0

0

0

0

10

10

10

0

0

0

0

0

0

0

0

0

148

116

0

0

0

0

0

0

0

0

0

0

1

1

0

0

0

0

0

0

0

0

0

0

0

0

0

0

0

0

55

0

0

0

55

0

5

1

0

0

1

0

0

0

0

3

0

0

0

44

44

10

1

9

1

0

0

0

1

9

9

9

0

0

0

14

0

0

0

14

14

0

0

0

0

0

0

0

0

0

0

0

0

0

0

0

0

9

1

0

1

0

0

0

0

0

0

0

0

6

1

5

0

0

0

0

0

0

0

0

0

2

1

1

0

0

0

0

0

0

0

0

7

0

0

0

0

0

0

7

7

7

0

0

0

0

0

0

3682

0

0

0

0

1249

346

0

0

0

0

0

0

6

0

5

0

0

1

32

5

27

233

233

45

45

24

24

6

6

903

13

13

0

0

0

9

9

881

0

0

0

140

0

9

161

0

0

11

536

0

0

0

9

9

6

0

0

0

0

0

0

0

0

0

0

0

0

0

158

37

0

0

0

0

34

34

1

0

0

0

0

1

0

0

0

0

0

1

1

0

1

1

121

12

12

58

58

4

2

2

1

0

1

25

23

2

0

0

0

0

1

1

3

0

3

5

5

11

11

0

0

1

1

0

0

0

0

0

0

0

0

0

0

60

60

1

1

0

0

59

4

55

0

0

0

0

0

0

0

0

0

0

463

463

0

0

0

0

417

72

137

0

0

2

0

0

9

0

0

0

0

5

30

102

0

0

0

6

4

0

19

13

18

0

0

0

0

18

18

28

0

16

12

0

0

0

0

0

0

0

0

0

0

0

0

0

1

1

1

1

0

1744

13

13

11

2

0

0

0

0

0

0

0

0

0

0

0

170

168

1

3

8

0

94

0

21

38

0

0

0

3

0

2

2

0

3

3

2

1

1558

156

156

66

66

10

5

1

4

1016

1

313

702

88

1

62

5

0

0

20

16

16

73

56

0

0

0

1

16

9

9

53

0

0

53

9

1

8

2

2

60

55

5

0

0

0

0

0

0

0

0

0

0

6

6

6

0

0

2

0

0

4

1

1

0

0

1

1

0

0

0

0

0

0

0

0

0

0

0

0

0

0

0

0

0

6

0

0

0

0

0

0

6

0

0

0

0

0

0

0

0

0

0

0

0

0

0

0

0

0

0

0

0

0

0

0

6

0

0

0

0

0

6

2

2

0

2

0

0

0

0

0

0

52

52

52

52

52

177

41

2

2

2

2

0

0

0

0

0

21

21

21

21

18

8

8

8

0

0

0

10

10

10

7

0

0

0

0

7

0

0

0

7

7

7

0

0

0

0

47

11

11

11

11

6

6

1

1

0

3

3

0

2

2

6

6

6

6

0

0

0

16

16

0

0

16

16

0

0

8

0

0

0

0

0

0

8

8

8

82

82

82

82

82

16

16

16

16

16

0

16

207

207

207

207

207

107

100

0

0

0

0

0

0

12

12

12

2

2

2

0

0

0

0

0

0

10

10

10

0

0

0

0

0

0

8

8

8

8

8

8

8

3

0

0

0

0

0

0

0

0

0

2

2

2

2

1

1

1

1

1

1

1

1

1

1

0

0

0

0

0

0
